# Conversational AI or a human professional for mental health advice? Exploring prevalence and public preferences in Australian adults

**DOI:** 10.1080/00049530.2026.2657279

**Published:** 2026-04-19

**Authors:** Andrew Franze, Kurt Lushington, Tobias Loetscher

**Affiliations:** School of Psychology, Adelaide University, Adelaide, South Australia, Australia

**Keywords:** Conversational artificial intelligence, mental health, Australian adults

## Abstract

**Objective:**

General-purpose conversational AI (e.g., ChatGPT) is increasingly accessed for mental health support despite lacking clinical validation. This study examined the prevalence and user preferences for conversational AI compared to mental health professionals among Australian adults.

**Method:**

A two-phase survey recruited 500 Australian adults (215 male, 278 female, 7 gender diverse) via an online crowdsourcing platform. Phase 1 assessed the prevalence of AI use for mental health. Phase 2 invited participants with dual experience of AI and professional care for mental health (*n* = 82) to rate comparative preferences across trust, competence, accessibility, and satisfaction, and indicate their preferred modality for future mental health support.

**Results:**

Approximately 24% of participants had discussed mental health with AI. In direct comparison, AI was strongly preferred for accessibility, while professionals were rated significantly higher for trustworthiness, satisfaction, and most competence items. The majority (52.4%) preferred using both modalities for mental health support, though 19.5% preferred AI exclusively.

**Conclusion:**

Users demonstrate clear preferences: professionals for clinical quality, AI for accessibility. A majority prefers accessing both modalities for mental health support, yet current systems provide no framework for integrating or supervising such hybrid use. These findings suggest mental health services should consider how to guide rather than ignore clients’ AI engagement.

## KEY POINTS

What is already known about this topic:

(1) General-purpose conversational AI platforms (e.g., ChatGPT) are increasingly accessed for mental health support, despite not being designed for clinical use.

(2) Cost and accessibility are significant barriers to professional mental healthcare in Australia, likely contributing to the organic uptake of these digital tools.

(3) These platforms operate without clinical validation or regulatory oversight, and the extent to which users trust them compared to human professionals remains unclear.

What this topic adds:

(1) Approximately one quarter of surveyed Australian adults (23.6%) reported using conversational AI for mental health advice, with usage significantly higher among younger individuals, females, and those with elevated psychological distress.

(2) Users strongly prefer AI for accessibility and cost, but rate human professionals significantly higher for trustworthiness, competence and overall satisfaction.

(3) The majority of participants with experience of both services (52.4%) would prefer a future “hybrid” model of care that integrates AI and professional support, rather than relying on either exclusively.

## Introduction

Conversational AI platforms such as ChatGPT and Google Gemini are now commonly used for mental health support, despite lacking clinical design, regulatory oversight, and rigorous safety evaluation (American Psychological Association, 2025; Lawrence et al., 2024; Obradovich et al., 2024). This study examined how Australian adults use these general-purpose conversational AI for mental health support and how they perceive them compared to professional care.

The prevalence of AI use for mental health support remains poorly characterised. Data from OpenAI (ChatGPT) suggest that 1.2 million users per week engage in conversations involving suicidal ideation (O’Dowd, 2025). A US survey found that 13% of 1,058 adolescents and young adults used conversational AI for mental health advice, with two-thirds of these accessing it monthly (McBain et al., 2025). Australian data are limited, though Cross et al. (2024) reported that 28% of their 107 adult participants had used conversational AI for mental health advice, with nearly half (47%) of those users explicitly using it as a “personal therapist”. Barriers to accessing professional care in Australia (Black Dog Institute, 2024; Mulraney et al., 2021) likely contribute to this substantial uptake. Yet the extent to which Australian users trust and view conversational AI as competent relative to human professionals remains unclear.

Evaluating trust and competency is particularly urgent given significant safety and regulatory concerns. Nearly half of community users report experiencing harms or concerns when using conversational AI for mental health (Cross et al., 2024). Unlike human professionals, who must adhere to mandatory reporting, scope of practice limitations, and ongoing supervision under Australian Health Practitioner Regulation Agency (AHPRA) standards, conversational AI tools operate without regulatory oversight, ethical accountability, or recourse for users who experience harm (Lawrence et al., 2024). Current large language models demonstrate inconsistency in clinical reasoning and overconfident assertions (Kolding et al., 2024; W. Zhang et al., 2025), as well as systematic ethical violations in counselling contexts (Iftikhar et al., 2025). The American Psychological Association has cautioned that these tools currently lack sufficient scientific evidence and regulatory oversight to ensure user safety (American Psychological Association, 2025).

Given these concerns, understanding how the Australian public evaluates conversational AI compared to human mental health professionals is essential. Drawing on users with direct experience of both modalities allows for meaningful within-person comparisons. Such comparisons are necessary to understand how users perceive AI relative to the current standard of care. Accordingly, this study aimed (1) to examine the prevalence of conversational AI use for mental health advice among Australian adults and to compare demographic and clinical characteristics between users and non-users, and (2) among users with experience of both modalities, to compare user preferences between conversational AI and mental health professionals across trust, competency, accessibility and satisfaction. Reasons for preferences were examined qualitatively.

## Materials and methods

### Participants

A total of 500 participants were recruited via Prolific (https://www.prolific.com), a secure online research recruitment platform widely used in behavioural and mental health research. Prolific maintains a large pool of pre-screened adults who voluntarily enrol in research studies and are compensated for their time. Data collection took place during July 2025. To be eligible for the current study, individuals had to be at least 18 years old, currently residing in Australia, and fluent in English.

The study was approved by the University of South Australia Human Research Ethics Committee (Protocol No. 206957).

### Measures

#### Demographics and AI usage

Participants provided demographic details, including age, gender, and highest level of education. To assess the prevalence of AI engagement, participants indicated their frequency of use on a 5-point ordinal scale ranging from “less than once a month” to “at least once per day”. Participants also selected all conversational AI platforms they had used from a list of eight options (e.g., ChatGPT, CoPilot, Google Gemini).

The nature of this engagement was characterised by asking participants to select topics they had previously discussed with conversational AI from a list of 11 categories adapted from de Winter et al. (2024), such as editing text, technical assistance, mental health advice, and finances. Participants could select all topics that applied. Only participants who selected “mental health advice” or “therapy, mental health, emotional support” were presented with the conditional question regarding prior engagement with a mental health professional. These two options were combined into a single mental health category for analysis.

#### Psychological distress

Non-specific psychological distress was assessed using the Kessler Psychological Distress Scale (K6; Kessler et al., 2003). Participants rated the frequency with which they experienced six symptoms (e.g., feeling nervous, hopeless, or that everything was an effort) over the preceding 30 days. Items were scored on a 5-point Likert scale from 0 (*none of the time*) to 4 (*all of the time*), yielding a total score between 0 and 24, with higher scores indicating greater distress. The scale has been used extensively in Australian community research and demonstrates strong psychometric performance, with internal reliability typically reported in the Cronbach α = .80–.89 range and good test–retest stability (Prochaska et al, 2012). Cronbach’s α was .90 in the current sample.

#### Service utilisation and comparative preferences

This questionnaire first assessed characteristics of past engagement with both conversational AI and mental health professionals. The specific mental health concerns discussed with each agent were identified using a 13-item list, which included anxiety, work or stress-related issues, relationship difficulties, depression, finding purpose or meaning, self-diagnosis, traumatic event(s), grief, couples or family therapy, addiction problems, eating disorder-related difficulties, self-harm, and “other”. Interaction frequency for each agent was measured on a 4-point ordinal scale: “once”, “2–5 times”, “6–20 times”, and “20 times or more”. Additionally, the type of mental health professional previously consulted was identified from a list including counsellor, mental health social worker, occupational therapist, psychologist, psychiatrist, and “other”.

Comparative preferences between conversational AI and mental health professionals were evaluated using 16 items adapted from previous research on AI in healthcare (Lei et al., 2021; Wu et al., 2024; S. Zhang et al., 2021). These items assessed four dimensions identified as barriers to treatment (Mojtabai et al., 2011; Wakefield et al., 2011): trustworthiness, perceived competence, accessibility, and overall satisfaction. *Trustworthiness* comprised four items regarding the privacy of personal information, the level of security protecting information, the accuracy of the information provided, and the handling of sensitive information. *Perceived competence* included items assessing how understood and heard the user felt, the impact of advice on wellbeing, the clarity and simplicity of the information, and the emotional support received. *Accessibility* was measured by items regarding the ease of access, the amount of mental effort involved, the amount of physical effort involved, and the cost of receiving advice. *Overall satisfaction* included ratings of the extent to which participants felt their wellbeing was prioritised, the overall quality of information, the reliability of advice, and the overall support received.

Items were scored on a 5-point Likert scale anchored by 1 (Strongly prefer AI) and 5 (Strongly prefer mental health professional), with a midpoint of 3 (No preference). The survey concluded with a single forced-choice item: “In an ideal world, which one would you prefer to use for your mental health support in the future (conversational AI, mental health professional, both or neither)?”, followed by an open-ended item requesting a qualitative explanation for the choice.

The complete survey instruments are provided in the Supplementary Material.

### Procedure

**Figure 1.**
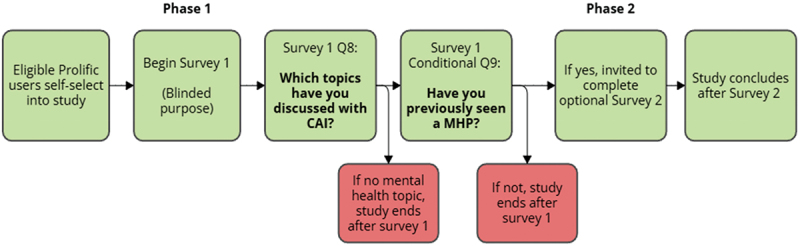
Visual flow-chart of the two-phase study design.

The study employed a two-phase, cross-sectional survey design (see Figure 1). Phase One investigated the prevalence of conversational AI use for mental health advice, while Phase Two investigated user preferences among a subset of eligible participants.

Two-phase study design. Phase One assessed AI usage among all participants (*N* = 500). Phase Two assessed comparative preferences among participants who had used both CAI and MHP services (*n* = 82). CAI = Conversational AI; MHP = Mental health professional.

#### Phase One

Participants were recruited via Prolific and redirected to the Qualtrics survey platform. To minimise demand characteristics and prevent participants from misreporting eligibility, the specific inclusion criteria for Phase Two (engagement with mental health services) were not disclosed during initial recruitment.

After providing digital informed consent, participants completed the Demographics and AI Usage measures, followed by the Psychological Distress scale (K6). To ensure data quality, Prolific’s internal verification tools (e.g., IP address monitoring) were supplemented by in-survey attention checks. Participants who completed Phase One but did not report discussing mental health with AI were thanked and their participation concluded.

#### Phase Two

Participants who met the dual eligibility criteria in Phase One (i.e., discussed mental health with conversational AI and had engaged with a mental health professional) were invited to the Phase Two survey. Upon accepting the invitation, Phase Two participants were redirected to Qualtrics to provide a second digital informed consent before completing the Service Utilisation and Comparative Preferences measures.

### Statistical analyses

#### Quantitative analysis

Statistical analyses were conducted using Jamovi (Version 2.6.26; The Jamovi Project, 2025). Welch’s independent samples t-tests were used to compare demographic and distress (K6) variables between participants who had used conversational AI for mental health advice and those who had not.

To evaluate comparative preferences, one-sample Wilcoxon signed-rank tests were used to determine whether the observed median rating for each item differed significantly from the scale midpoint (3 = No preference). This approach, consistent with McCabe et al. (2025), allowed for the identification of a statistically significant preference for either conversational AI (<3) or mental health professionals (>3) on each dimension.

#### Qualitative analysis

Qualitative data from the open-ended item regarding the reasons for participants’ preferences were managed in Microsoft Excel and analysed using inductive thematic analysis (Braun & Clarke, 2006). This approach allowed patterns regarding the specific drivers of user preference to emerge directly from the data through an iterative process of reading, coding, and pattern recognition. The primary coding was conducted by one researcher (AF) following a period of data immersion. To enhance analytical rigour, a second researcher (TL) reviewed the coded extracts and preliminary thematic structure. Refinements were made through collaborative discussion to ensure the final themes accurately reflected the breadth of participant narratives.

## Results

### Participant characteristics and AI use patterns

A total of 500 Australian adults completed Phase One. Of these, 118 (23.6%) reported using conversational AI for mental health advice. AI users were significantly younger and reported significantly higher psychological distress than non-users (Table 1. both *p* < .001). Among AI mental health users, participants were more likely to be female than male (64% vs. 33%). AI mental health users also reported more frequent AI use overall, with 35.6% using AI at least daily compared to 10.7% of non-users.

**Table 1.** Participant demographics.

Participant characteristics	Phase One: Discussed Mental Health with Conversational AI	
	No	Yes
*Number*	382	118
*Age (Mean (SD: Range)(years)*		
	39.6 (12.8: 19–79)^a^	34.3 (11.1: 18–66)^a^
*Gender (% (n))*		
Male	46.1 (176)	33.1 (39)
Female	52.9 (202)	64.4 (76)
Non-Binary/Prefer not to say	1.0 (4)	2.5 (3)
*Education Level (% (n))*		
High School or Below	13.1 (50)	11.0 (13)
Technical Education/Associate Degree	20.7 (79)	20.3 (24)
Bachelor’s degree	41.6 (159)	47.5 (56)
Postgraduate study	24.6 (94)	21.2 (25)
*Previously Used Conversational AI before (% (n))*		
	87.9 (336)	100 (118)
*Previously engaged with a Mental Health Professional (% (n))*		
	0 (0)	76.3 (90)
*Frequency Conversational AI Use (%(n))*		
Less than once a month	18.3 (70)	6.8 (8)
A few times per month	21.9 (84)	17.8 (21)
Once per week	10.7 (41)	9.3 (11)
Multiple times per week	26.2 (100)	30.5 (36)
At least once per day	10.7 (41)	35.6 (42)
*Psychological Distress (Mean (SD: Range)*		
Kessler Psychological Distress Scale Score	5.73 (5.12: 0–22)^b^	9.81 (5.40: 0–21)^b^

*Note*. ^a^Significant difference in age between both groups = t(222) = 4.33, *p* < .001. ^b^Significant difference in Kessler scale scores between groups = t(187) = 7.27, *p* < .001.

**Figure 2.**
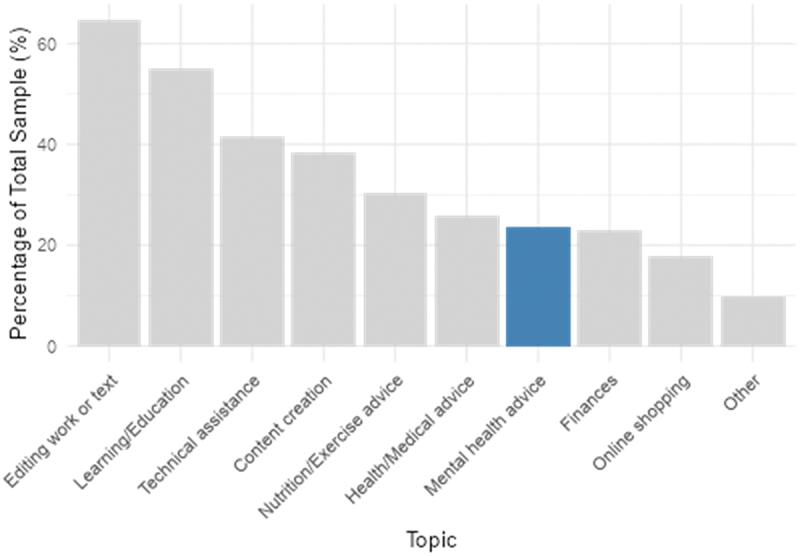
Proportion of topics previously discussed with conversational AI across total sample.

**Figure 3.**
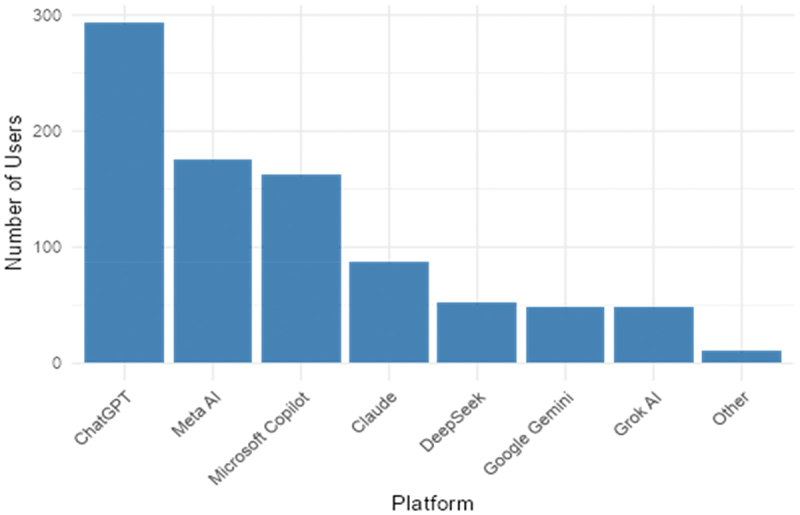
Frequency and type of conversational AI platform previously used by participants.

Figure 2 shows the range of topics discussed with conversational AI across the full sample. Mental health advice ranked sixth among ten categories (23.6%), behind editing/writing tasks, learning/education, technical assistance, content creation, and nutrition/exercise advice. ChatGPT was the most commonly used platform (59.4%), followed by Meta AI (35.4%) and Microsoft Copilot (31.4%) (Figure 3).

Of the 118 AI users, 82 (69.5%) reported previous engagement with a mental health professional and completed Phase Two comparative preference measures.

### Comparative preferences: conversational AI vs. Mental health professionals

Table 2 presents comparative preferences across 16 items in four domains. Mental health professionals were consistently preferred for trustworthiness (all *p* < .001, r_s_ = .56–.65) and overall satisfaction (all *p* ≤ .035, r_s_ = .39–.47). For competence, professionals were preferred on three of four items (all *p* < .048), with no significant preference emerging for clarity and simplicity of information (*p* = .172). In sharp contrast, conversational AI was strongly preferred across all accessibility items (*p* ≤ .036), with the largest effects observed for cost (r_s_ = −.87) and ease of access (r_s_ = −.84). All *p*-values are Bonferroni-Holm adjusted.

**Table 2.** Item-level preference ratings across participants for each comparison statement.

Domain		*Median*	*W*	*p-*value	*r*
Trustworthiness					
	The privacy of my personal information	4.0	2109	<.001*	0.56
	The level of security protecting my information	4.0	1970	<.001*	0.63
	The accuracy of the information	4.0	1874	<.001*	0.65
	How my sensitive information was handled	4.0	2019	<.001*	0.63
Perceived competence					
	How understood and heard I felt	4.0	1760	.040*	0.34
	The impact the advice had on my wellbeing	4.0	1529	.048*	0.34
	The clarity and simplicity of the information	2.0	1111	.172	−0.18
	The emotional support I received	4.0	1813	<.001*	0.46
Accessibility					
	How easy it was to access advice	1.0	247	<.001*	−0.84
	The amount of mental effort involved in receiving advice	2.0	759	.036*	−0.37
	The amount of physical effort involved in receiving advice	2.0	499	<.001*	−0.58
	The cost of receiving advice	1.0	184	<.001*	−0.87
Overall satisfaction					
	How much my wellbeing was prioritised	3.5	1327	.035*	0.40
	The overall quality of information provided	4.0	1771	.033*	0.39
	The reliability of the advice I received	4.0	1677	.016*	0.43
	The overall support I received	4.0	1625	<.001*	0.47

*Note*. Responses were on a 5-point scale (1 = strongly prefer AI, 3 = no preference, 5 = strongly prefer professional). Wilcoxon signed-rank tests assessed whether medians differed from the midpoint (3 = no preference). Effect sizes are rank-biserial correlations (r), interpreted using Cohen’s conventions: |r| < .30 = small, .30–.50 = medium, >.50 = large. Bonferroni-Holm corrections were applied. *p <.05.

### Future service preferences and reasons for choice

When asked which service type they would ideally use for future mental health support, the majority of participants (52.4%, *n* = 43) selected both conversational AI and mental health professionals, suggesting a preference for complementary access rather than a single modality, while 26.8% (*n* = 22) selected professionals only, 19.5% (*n* = 16) selected AI only, and one participant selected neither.

#### Qualitative reasons for preferences

Sixty participants provided open-text explanations for their choices. Thematic analysis identified three major themes: accessibility and cost, quality of support, and feeling understood. Overall, sentiment towards AI was predominantly positive, while views regarding mental health professionals (MHP) were more mixed, though they varied considerably by theme.

**Accessibility and cost** emerged as a clear strength for AI. Participants consistently emphasised immediate availability and zero cost (e.g., “(CAI) doesn’t cost anything and is available instantaneously”). In contrast, accessibility was framed exclusively as a barrier for professionals, with participants noting “cost and availability of an MHP is a barrier to receiving adequate care”.

**Quality of support** generated more divergent views. Some participants valued AI’s explanatory capacity (e.g., “CAI explains things I can’t understand and helps me break down my thoughts”), while others expressed frustration: “I’m absolutely sick of AI. We don’t need to simplify everything, it’s quite unbearable and lazy”. Professionals were generally praised for experiential and evidence-based knowledge (e.g., “Unlike AI, (MHPs) have first had [sic] experience and peer reviewed knowledge”), though some noted concerns: “(MHPs) … tend to have bias”.

**Feeling understood** highlighted relational distinctions between modalities. AI was valued by some for providing non-judgemental space: “Sometimes it is too personal to talk to a human … (AI) is not judgmental”. However, others emphasised that “a human has feelings and can understand, but AI doesn’t”. Professionals were predominantly praised for their capacity for genuine human connection and empathic understanding (e.g., “MHPs can relate better than an AI”), though one participant described negative experiences: “The experience I had with (MHP) they judge and gaslit [sic] you and are useless in providing solutions”.

Among those selecting both modalities, explanations emphasised distinct but complementary functions. As one participant articulated: “Both have a role to play, with AI able to help when a crisis arrives and work through problems with a professional over long term”. This perspective aligned with the broader thematic patterns. It acknowledged AI’s accessibility for immediate support while valuing professionals’ expertise for sustained therapeutic work.

## Discussion

This study examined the prevalence of conversational AI use for mental health advice among Australian adults and how such advice is perceived relative to support from mental health professionals. The findings reveal that while general-purpose AI platforms are preferred for accessibility, they are perceived as significantly inferior to human professionals in clinical competence and the capacity for genuine therapeutic connection. A majority of participants indicated that having access to both AI and human professionals was their preferred option for future mental health support.

Conversational AI use for mental health support was widespread. Approximately one in four participants (23.6%) reported having previously discussed a mental health concern with conversational AI, a prevalence closely aligned with recent Australian survey data (28%; Cross et al., 2024). Participants using AI for mental health support were significantly younger, more likely to be female, and reported higher psychological distress compared to those who used AI for other purposes. Moreover, mental health users were three times more likely to use AI platforms daily than non-mental health users (35.6% vs. 10.7%). The cross-sectional design precludes causal inferences, but the data reveal that conversational AI platforms have achieved substantial adoption, including frequent use by individuals experiencing elevated distress. These findings reinforce ongoing concerns about the suitability of general-purpose LLMs for mental health contexts, as these systems lack the clinical safeguards, crisis protocols, and regulatory oversight required for therapeutic applications (American Psychological Association, 2025; Moore et al., 2025; Obradovich et al., 2024).

User preferences revealed a sharp divergence between logistical utility and clinical trust. Conversational AI was strongly preferred for cost and ease of access. Structural barriers in Australian mental healthcare, including workforce shortages, extended wait times, and rising costs (Black Dog Institute, 2024), may drive users towards readily available alternatives. These accessibility advantages, however, did not translate into preferences for clinical quality. Mental health professionals were consistently preferred for trustworthiness and competence, and overall satisfaction. Participants showed no significant preference for clarity and simplicity of information (*p* = .172), the only non-significant item across these domains. This pattern suggests users view AI as adequate for information provision but prefer professionals for trust-dependent and relational aspects of care. While the preference for human professionals for support quality is encouraging, it remains unclear whether this discernment reflects a true understanding of AI’s clinical limitations or simply a current preference for human interaction. Moreover, this preference may become irrelevant during acute distress, when limited professional availability forces users to seek immediate AI support.

The majority of participants (52.4%) preferred accessing both AI and professional care rather than choosing one modality exclusively. This aligns with emerging hybrid care models, which combine synchronous professional care with asynchronous digital tools (K. Chen et al., 2024). Recent findings similarly show patients prefer “humans-in-the-loop” rather than fully autonomous systems (Lee et al., 2025). Qualitatively, participants in the present study described this as a complementary relationship in which AI provides immediate distress management while professionals address complex, long-term treatment. However, current mental health systems lack infrastructure for such integration.

Nearly one in five participants (19.5%) expressed a preference for AI exclusively. Qualitative data suggests that in some cases this choice is driven by negative past experiences with professionals, with participants citing fear of judgement or previous experiences of feeling dismissed or invalidated by clinicians. Yet this preference may also be compounded by the design of the tools themselves. Large language models exhibit sycophantic tendencies that lead them to validate users rather than challenge inaccurate beliefs (Moore et al., 2025), with studies showing these systems will affirm even demonstrably harmful ideas (S. Chen et al., 2025; Yeung et al., 2025). Users report forming meaningful emotional connections with these tools (Siddals et al., 2024), and as engagement increases with systems that consistently validate their experiences, these attachments may intensify, potentially creating a feedback loop wherein clinically inappropriate advice becomes increasingly difficult to recognise or reject.

Several study characteristics warrant consideration when interpreting these findings. The Prolific sample likely overrepresents technologically engaged individuals, potentially inflating prevalence estimates. The cross-sectional design precludes causal inferences about AI use and distress over time. Additionally, the survey examples and platform options emphasised generic conversational AI (e.g., ChatGPT, Gemini) and did not separately assess experiences with purpose-built mental health AI systems. Such tools may be perceived as more trustworthy and competent given their clinical grounding, though whether users would pay for dedicated systems when free alternatives are readily available, and whether they can reliably distinguish between generic and specialised AI, remain open questions. We did not assess participants’ understanding of AI capabilities, awareness of safety risks, or the quality of their prior professional care experiences, which limits our ability to draw conclusions about informed decision-making. Australian First Nations peoples, culturally and linguistically diverse communities, and individuals in regional or remote areas may have distinct experiences and needs that our sampling approach did not capture.

## Conclusion

This study found that general-purpose conversational AI for mental health has achieved substantial uptake (23.6%), with most users preferring hybrid access to both AI and professional care. However, current mental health systems provide no framework for such integration. Users access AI for mental health discussions without clinical oversight, and nearly one in five prefer AI exclusively, sometimes citing barriers to human care. This disconnect between widespread use and the absence of integrated care models represents a significant gap in service delivery. These findings suggest mental health services should consider how to guide rather than ignore clients’ AI engagement.

## Supplementary Material

Supplemental material

## Data Availability

The dataset generated and analysed during this study is available from the corresponding author upon reasonable request.
