# Supplementary material for: Conversational AI or a human professional for mental health advice? Exploring prevalence and public preferences in Australian adults
Source: Aust J Psychol. 2026 Apr 19;78(1):2657279. doi: 10.1080/00049530.2026.2657279 (PMC13094287; doi:10.1080/00049530.2026.2657279)
Supplement: Supplemental material [file RAUP_A_2657279_SM3999.pdf]

Supplementary Material

Conversational AI or a human professional for mental health advice? Exploring prevalence and public preferences in Australian Adults

Andrew Franze[1], Kurt Lushington[1], Tobias Loetscher[1]

[1]*School of Psychology, Adelaide University, South Australia, Australia*

Corresponding Author
Tobias Loetscher, School of Psychology, Adelaide University, South Australia, Australia,
email: tobias.loetscher@adelaide.edu.au

Survey 1: Exploring Prevalence and Topics Discussed with Conversational AI in the Australian Public

Start of Block: Screener Validation

Q1 What is your country of Residence?

- Australia (1)
- New Zealand (2)
- United States (3)
- United Kingdom (4)
- Other (5)

End of Block: Screener Validation

Start of Block: Age Screener, 18+

*

Q2 What is your age?

____

End of Block: Age Screener, 18+

Start of Block: Default Question Block

Q3 What is your gender?

- Male (1)
- Female (2)
- Non-binary / third gender (3)
- Prefer not to say (4)

Q4 What is your highest level of education completed?

- ○ Below high school (1)
- ○ High school or equivalent (2)
- ○ Technical and further education or associate degree (3)
- ○ Bachelor degree (4)
- ○ Postgraduate degree or PhD (5)

Page Break

Q5 Have you ever used Conversational AI before? (E.g. ChatGPT, Gemini, Claude, Grok)

- Yes (1)
- No (2)
- Not sure (3)

Display this question:

If Have you ever used Conversational AI before? (E.g. ChatGPT, Gemini, Claude, Grok) = Yes

Q6 Which Conversational AI platform have you used? (select all that apply)

- ChatGPT (1)
- Claude (2)
- Google Gemini (3)
- DeepSeek (4)
- Meta AI (5)
- Microsoft Copilot (6)
- Grok AI (7)
- Other, please list: (8) ____

Display this question:

If Have you ever used Conversational AI before? (E.g. ChatGPT, Gemini, Claude, Grok) = Yes

Q7 How often do you use Conversational AI? (E.g. ChatGPT, Gemini, Claude, Llama)

- ○ Less than once per month (1)
- ○ A few times per month (2)
- ○ Once per week (3)
- ○ Multiple times per week (4)
- ○ At least once per day (5)

Page Break

Display this question:

If Have you ever used Conversational AI before? (E.g. ChatGPT, Gemini, Claude, Grok) = Yes

Q8 Which topics have you spoken to Conversational AI about? (Select all that apply)

- ☐ Editing work or text (1)
- ☐ Finances (2)
- ☐ Nutrition or Exercise advice (3)
- ☐ Mental health advice (4)
- ☐ Online shopping (5)
- ☐ Therapy, mental health, emotional support (6)
- ☐ Content creation (7)
- ☐ Learning and education (8)
- ☐ Health and medical advice (9)
- ☐ Technical assistance (e.g., coding help, troubleshooting, IT support) (10)
- ☐ Other, please list: (11) ____

Page Break

Display this question:

If Which topics have you spoken to Conversational AI about? (Select all that apply) = Mental health advice

Or Which topics have you spoken to Conversational AI about? (Select all that apply) = Therapy, mental health, emotional support

Q9 Have you ever seen a health professional for mental health concerns?

- ◯ Yes (1)
- ◯ No (2)
- ◯ Not sure (3)

Page Break

Display this question:

If Have you ever seen a health professional for mental health concerns? = Yes

Q10 I give permission to be contacted for a follow-up survey on this topic:

◯ Yes (1)

◯ No (2)

End of Block: Default Question Block

Start of Block: K6

K6 We would also like to know about your overall level of psychological wellbeing **over the last month**. Please answer the following 6 questions. ***Over the last 30 days, about how often did you feel...***

	None of the time (0)	A little of the time (1)	Some of the time (2)	Most of the time (3)	All of the time (4)
...nervous? (4)	◯	◯	◯	◯	◯
...hopeless? (5)	◯	◯	◯	◯	◯
...restless or fidgety? (6)	◯	◯	◯	◯	◯
...so sad that nothing could cheer you up? (7)	◯	◯	◯	◯	◯
...that everything was an effort? (8)	◯	◯	◯	◯	◯
...worthless? (9)	◯	◯	◯	◯	◯

End of Block: K6

Survey 2: Exploring Prevalence and User Preferences for Seeking Mental Health from Conversational AI Compared to Mental Health Professionals

Start of Block: Default Question Block

Q1 In regards to your mental health, which topics have you sought advice on from Conversational AI? (select as many as apply)

- ▢ Self-Diagnosis (1)
- ▢ Relationship difficulties (2)
- ▢ Anxiety (3)
- ▢ Depression (4)
- ▢ Grief (5)
- ▢ Self-harm (6)
- ▢ Finding purpose or meaning (7)
- ▢ Traumatic event/s (8)
- ▢ Couples or family therapy (9)
- ▢ Difficulties related to an eating disorder (10)
- ▢ Problems with addictions (e.g. gambling or alcohol use) (11)
- ▢ Work or stress related issues (12)
- ▢ Other (please describe as much as you feel comfortable) (13) ____

Page Break

Q2 How many times in total have you spoken to Conversational AI about this concern/s?

- Once (1)
- 2 to 5 times (2)
- 6 to 20 times (3)
- 20 times or more (4)

Q3 Which type of mental health professional have you spoken to before? (select as many as apply)

- Counsellor (1)
- Mental Health Social Worker (2)
- Occupational Therapist (3)
- Psychologist (4)
- Psychiatrist (5)
- Other, please list: (6) ____

Page Break

Q4 Which topics did you discuss with the mental health professional? (select as many as apply)

- ☐ Mental Health Diagnosis (1)
- ☐ Relationship difficulties (2)
- ☐ Anxiety (3)
- ☐ Depression (4)
- ☐ Grief (5)
- ☐ Self-harm (6)
- ☐ Finding purpose or meaning (7)
- ☐ Traumatic event/s (8)
- ☐ Couples or family therapy (9)
- ☐ Difficulties related to an eating disorder (10)
- ☐ Problems with addictions (e.g. gambling or alcohol use) (11)
- ☐ Work or stress related issues (12)
- ☐ Other (please describe as much as you feel comfortable) (13) ____

Q5 How many times in total have you spoken to a mental health professional about this concern/s?

- Once (1)
- 2 to 5 times (2)
- 6 to 20 times (3)
- 20 times or more (4)

Page Break

Intro Comparing your experiences in discussing mental health, rate your preference between Conversational AI and a human Mental Health Professional (MHP) for each of the next 17 statements below:

Page Break

JS X→

Trust

	Strongly Prefer AI (1)	Slightly Prefer AI (2)	No Preference (3)	Slightly Prefer a MHP (4)	Strongly Prefer a MHP (5)
1. I preferred the privacy of my personal information with... (18)	○	○	○	○	○
2. I preferred the level of security protecting my information from... (19)	○	○	○	○	○
3. I preferred the accuracy of the information provided by... (20)	○	○	○	○	○
4. I preferred how my sensitive information was handled by... (21)	○	○	○	○	○

Page Break

JS

Perceived Competence

	Strongly Prefer AI (1)	Slightly Prefer AI (2)	No Preference (3)	Slightly Prefer a MHP (4)	Strongly Prefer a MHP (5)
5. I preferred how understood and heard I felt with... (50)	○	○	○	○	○
6. I preferred the impact the advice had on my wellbeing from... (51)	○	○	○	○	○
7. I preferred the clarity and simplicity of the information from... (52)	○	○	○	○	○
8. I preferred the emotional support I received from... (53)	○	○	○	○	○

JS X→

Attention Item

	Strongly Prefer AI (0)	Slightly Prefer AI (1)	No Preference (0)	Slightly Prefer a MHP (0)	Strongly Prefer a MHP (0)
9. Please answer this item as 'Slightly Prefer AI' - this is a test of your attention. (50)	○	○	○	○	○

Page Break

JS

Accessibility

	Strongly Prefer AI (1)	Slightly Prefer AI (2)	No Preference (3)	Slightly Prefer a MHP (4)	Strongly Prefer a MHP (5)
10. I preferred how easy it was to access advice from... (54)	○	○	○	○	○
11. I preferred the amount of mental effort involved in receiving advice from... (55)	○	○	○	○	○
12. I preferred the amount of physical effort involved in receiving advice from... (56)	○	○	○	○	○
13. I preferred the cost of receiving advice from... (57)	○	○	○	○	○

Page Break

JS

Satisfaction

	Strongly Prefer AI (1)	Slightly Prefer AI (2)	No Preference (3)	Slightly Prefer a MHP (4)	Strongly Prefer a MHP (5)
14. I preferred how much my wellbeing was prioritised by... (45)	○	○	○	○	○
15. I preferred the overall quality of information provided by... (46)	○	○	○	○	○
16. I preferred the reliability of the advice I received from... (47)	○	○	○	○	○
17. I preferred the overall support I received from... (48)	○	○	○	○	○

Page Break

Q6 In an ideal world, which one would you prefer to use for your mental health support in the future?

- ○ Conversational AI (1)
- ○ Mental health professional (2)
- ○ Both of the above (3)
- ○ Neither (4)

Q7 Please feel free to further explain your answer to the previous question (Q6)

____

____

____

____

____

End of Block: Default Question Block
